# Using ultra-sensitive next generation sequencing to dissect DNA damage-induced mutagenesis

**DOI:** 10.1038/srep25310

**Published:** 2016-04-28

**Authors:** Kaile Wang, Xiaolu Ma, Xue Zhang, Dafei Wu, Chenyi Sun, Yazhou Sun, Xuemei Lu, Chung-I Wu, Caixia Guo, Jue Ruan

**Affiliations:** 1 Key Laboratory of Genomics and Precision Medicine, Beijing Institute of Genomics, Chinese Academy of Sciences, Beijing 100101, China; 2 University of Chinese Academy of Sciences, Beijing, China; 3 State Key Laboratory of Biocontrol, School of Life Sciences, Sun Yat-Sen University, Guangzhou, China; 4 Department of Ecology and Evolution, University of Chicago, USA; 5 Agricultural Genomics Institute at Shenzhen, Chinese Academy of Agricultural Sciences, Shenzhen, China

## Abstract

Next generation sequencing (NGS) technologies have dramatically improved studies in biology and biomedical science. However, no optimal NGS approach is available to conveniently analyze low frequency mutations caused by DNA damage treatments. Here, by developing an exquisite ultra-sensitive NGS (USNGS) platform “EasyMF” and incorporating it with a widely used supF shuttle vector-based mutagenesis system, we can conveniently dissect roles of lesion bypass polymerases in damage-induced mutagenesis. In this improved mutagenesis analysis pipeline, the initial steps are the same as in the supF mutation assay, involving damaging the pSP189 plasmid followed by its transfection into human 293T cells to allow replication to occur. Then “EasyMF” is employed to replace downstream MBM7070 bacterial transformation and other steps for analyzing damage-induced mutation frequencies and spectra. This pipeline was validated by using UV damaged plasmid after its replication in lesion bypass polymerase-deficient 293T cells. The increased throughput and reduced cost of this system will allow us to conveniently screen regulators of translesion DNA synthesis pathway and monitor environmental genotoxic substances, which can ultimately provide insight into the mechanisms of genome stability and mutagenesis.

Genomic DNA is constantly endangered by a variety of exogenous and endogenous agents, including ultraviolet (UV) radiation, X-ray, and chemical reagents. Usually the majority of DNA lesions can be recognized and repaired by different DNA repair pathways. However, some may escape the surveillance of cellular repair machinery and persist during S-phase, interfering with DNA replication and cell viability. To reduce this potential threat, cells have evolved a translesion DNA synthesis (TLS) system to replicate unrepaired DNA damages^1^. Multiple specialized DNA polymerases, including Polη and REV1, are utilized in TLS pathway^1^,^2^. Polη is encoded by *POLH* gene in human, which is specifically required for error-free bypass of cyclobutane pyrimidine dimers (CPDs) in DNA generated by exposure of cells to UV radiation. Inactivation of Polη renders cells hypermutable after UV radiation. Mutations in the *POLH* gene result in a variant form of the human genetic disorder xeroderma pigmentosum (XPV), a disease characterized by extreme sunlight sensitivity and an early predisposition to skin cancer. REV1 is another TLS polymerase, which mainly functions as a scaffold protein for polymerase switching at a lesion site due to its C-terminal region interacting with multiple specialized DNA polymerases implicated in TLS^3^,^4^,^5^,^6^. REV1 is believed to play a critical role in DNA damage-induced nucleotide substitutions in eukaryotes^2^,^7^,^8^. Determination of DNA damage-induced mutation characters is essential for comprehensively understanding TLS pathway regulation.

supF shuttle vector-based mutagenesis assay^9^ is widely used to measure the effects of lesion bypass DNA polymerases on damage-induced mutagenesis in mammalian cells^10^. However, given that a large number of transformed MBM7070 colonies are required to fulfill that experiment, this process is quite laborious and time-consuming. Additionally, since only surviving clones are enumerated for mutation frequency determination and just partial mutant clones are sequenced for mutation spectrum depiction, the final result is easily biased. Moreover, the mutation spectra based on the 95 bp of pSP189 plasmid could not represent the whole 5 kb plasmid comprehensively.

Next generation sequencing (NGS) technologies have dramatically improved researches in biology and biomedicine. However, an inevitable error rate of NGS approaches resulted from library preparation and sequencing remains high^11^, ranging from 0.1% to 1% at disparate platforms^12^,^13^,^14^ and data processing strategies^14^,^15^. It severely obscures the precise determination of mutations whose frequencies are almost lower than 1%. Fortunately, great efforts have been substantially made to develop exquisite ultra-sensitive next generation sequencing (USNGS) approaches for addressing this problem^16^,^17^,^18^,^19^,^20^,^21^,^22^,^23^,^24^,^25^,^26^,^27^,^28^,^29^. Majority of the USNGS methods, such as Safe-seq and Duplex-seq, utilize unique barcodes (or tags) to eliminate PCR and sequencing errors^20^,^27^,^28^,^29^,^30^. However, the efficiency of these methods relies heavily on the read number of each “read family”. To attain a high-precision result, one molecule should be sequenced many times, which severely constrains the efficiency of reads utility. The other method, circle sequencing (Cir-seq), tandems the replicates of one single-strand circularized molecule by rolling circle amplification (RCA)^18^,^22^ to achieve a tag-free read family. The original molecule can be sequenced at least twice by a pair of pair end (PE) reads through controlling the initial DNA fragment lengths, which effectively surmounted the disadvantage of the barcode methods. However, the effectivity of Cir-seq in dissecting low frequency mutation from low input DNA is not well testified.

In this study, we improved the Cir-seq experimental procedures and developed a highly efficient, easy-to-use data processing pipeline to identify the low frequency mutations from low input DNA (from dozens to hundreds nanogram) on Illumina HiSeq platforms. Based on this, we integrated the entire protocols into a package entitled “Easy Mutation Frequency detection platform” (“EasyMF”), and used this package to dissect *in vivo* roles of TLS polymerases in UV-induced mutagenesis. We measured the mutation frequencies of UV damaged plasmids (220 J/m^2^) in control, REV1 knocked down (6.25E-05) and Polη knocked down (1.26E-04) 293T cells. We noticed that over 99% minor allele frequency (MAF) were lower than 0.1%, among which three types of frequencies, 0, 0.00001 ~ 0.0001 and 0.0001 ~ 0.001 dominated the results. The mutation frequencies of all types of mutation (n = 12) increased significantly *(p* < *0.05)* after UV radiation in tested cells. In line with previous studies^10^,^31^,^32^,^33^, the mutation frequency of UV damaged plasmids in control cells was significantly lower than that in Polη knocked down cells (p < 0.05), while it is remarkably higher than that in REV1 knocked down cells (p < 0.05). Our data also indicated the mutation frequencies of A = >T, T = >A and G = >T were more significantly increased (*p* < *0.01*), while those of three other types of mutation (A = >C, C = >T, T = >C) exhibited no remarkable change *(p* > *0.05)* in Polη knocked down cells. In contrast, the mutation frequencies of two types of mutation (G = >T, C = >A) manifested a highly significant decrease (*p* < *0.01*), accompanied by an unapparent change of three types of mutation (A = >C, C = >T, G = >A) *(p > 0.05)* in the REV1 knocked down cells. We found there were some mutation hotspots (such as regions spanning 800 ~ 900 bp, 1.6 ~ 1.7 kbp, 2.4 ~ 2.5 kbp, 3.4 ~ 3.5 kbp) and conserved regions (500 ~ 600 bp, 1.4 ~ 1.5 kbp, 3.6 ~ 3.7 kbp) in the pSP189 plasmid. The sampled sequencing data indicated that even 50 Mega base (Mb) data would be enough to detect the distinct effects of TLS polymerases on UV-induced mutagenesis, which means the sequencing cost would be very low. Therefore, we have established a general experimental and data processing platform for rapid characterization of mutations on the supF shuttle vector in mammalian cells, which benefits from increased throughput and reduced costs.

## Results

### “EasyMF” pipeline for characterization of DNA damage-induced mutation frequency and spectra

**Figure 1:**
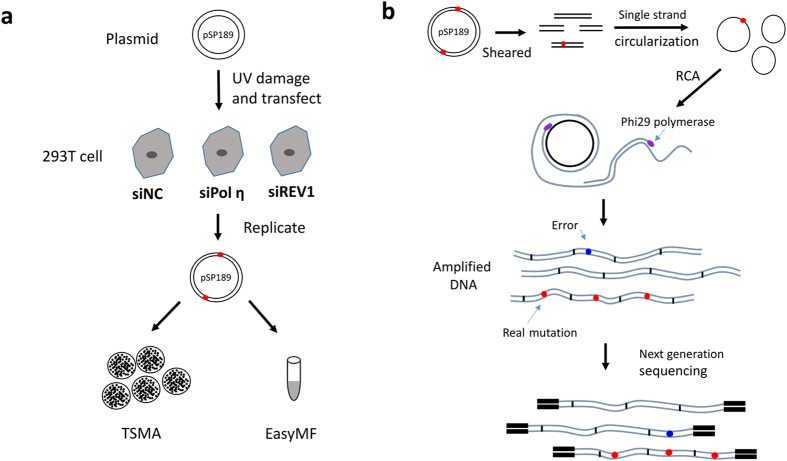
DNA damage-induced mutagenesis and EasyMF sequencing library preparation. **(a)** Schematic diagram of mutation frequency analysis in pSP189 using traditional supF shuttle vector-based mutagenesis assay (TSMA) and “EasyMF”. The undamaged or damaged (220 J/m^2^ UVC) pSP189 plasmid was transfected into the indicated siRNA-treated 293T cells. Mutation frequency in pSP189 was determined after it was recovered from 293T cells through both traditional supF shuttle vector-based mutagenesis assay (TSMA) and “EasyMF”. **(b)** The diagram of “EasyMF”. DNA was sheared into fragments shorter than half the length of the sequencing read and circularized, then used for RCA. Next, the amplified DNA were taken to prepare standard NGS libraries. The real mutation will appear in each tandem repeats (red dots), conversely, errors resulted from the RCA or NGS library preparation only happen in some of repeats (blue dots) randomly. Since the size of sheared DNA fragments shorter than the length of a single PE read, the original DNA will be sequenced at least twice in a pair of PE reads independently to eliminate PCR and sequencing errors. The consensus sequence (CS) can be determined by align read 1 and read 2 in one pair of PE reads, and the site of CS will be given with high quality scores if it is supported by at least twice, and it will be given with the lowest quality scores if it is supported by only once.

Mutagenesis assay is essential for measurement of TLS activity in mammalian cells^10^. However, no optimal NGS approach is available to conveniently analyze low frequency mutations caused by DNA damage treatments. To meet that challenge, we first optimized the Cir-seq experimental procedures to fulfill the low input requirements, and simplified the data process by providing high efficient and easy-to-use scripts. The pipeline, named as “EasyMF”, was used to distinguish the slight variations in mutation frequency between control and Polη or REV1 knocked down cells (Figs 1a and S1). The undamaged and UV damaged pSP189 plasmids were transfected into control, Polη or REV1 knocked down 293T cells, in which the plasmids were replicated. The amplified plasmids were then isolated and undergone traditional supF shuttle vector-based mutagenesis (TSMA) or “EasyMF” assay. Fig. 1b depicts how “EasyMF” filters sequencing errors and detects low frequency mutations. Briefly, the DNA was sheared into small fragments shorter than the length of a single PE read, denatured into single-strand molecules followed by circularization with single-strand DNA ligase. The circularized single-strand DNA fragments were used to perform rolling circle amplification (RCA). After amplification, the purified DNA were taken to prepare the standard Illumina HiSeq libraries. This method will ensure that different copies of each fragment could be sequenced at least twice in a pair of PE reads for error correction, to eliminate PCR and sequencing errors.

### Characterization of UV-induced mutation frequency and spectra with “EasyMF”

**Figure 2:**
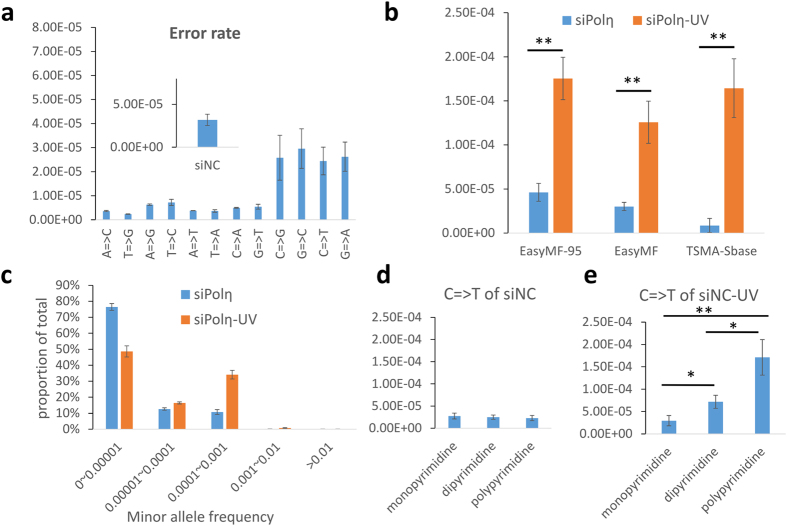
Mutation frequency pattern in DNA damage-induced mutagenesis measured by “EasyMF”. **(a)** Background mutation frequency and mutation spectrum of “EasyMF” in undamaged pSP189 from control cells. **(b)** Mutation frequencies in undamaged and UV damaged pSP189 plasmids were determined after they were recovered from siPolη-treated 293T cells through “EasyMF”, “EasyMF95” (mutation frequency of every base in 95bp target region), TSMA-Sbase (mutation frequency of single base measured by TSMA). siPolη and siPolη-UV represent undamaged or UV damaged plasmid transfected into siPolη-treated 293T cells, respectively, *0.01 = <p < 0.05, **p < 0.01. **(c)** Distribution of mutations’ MAFs of undamaged and UV damaged pSP189 plasmids in siPolη-treated 293T cells, y axis represents the proportion of mutations, and x axis represents interval of MAFs. **(d**) C = >T mutation frequencies at monodipyrimidine, dipyrimidine and polypyrimidine sites of undamaged plasmid in control 293T cells. **(e)** C = >T mutation frequencies at monodipyrimidine, dipyrimidine and polypyrimidine sites of UV damaged plasmid in control 293T cells.

We first evaluated the background mutation frequency of “EasyMF” in our experimental circumstance, which was calculated as the fraction of identified consensus bases (see methods) that differed from the reference sequence. We analyzed the sequencing data of undamaged plasmids from control cells, the results indicated “EasyMF” had an average mutation frequency of 3.19E-05(±6.57E-06). Notably, although most types of mutation had frequency lower than 1E-05, four types of mutation (C = >G, G = >C, C = >T and G = >A) displayed higher mutation frequencies and greater variance (Fig. 2a).

To examine whether “EasyMF” is sensitive enough to detect DNA damage-induced mutagenesis, we transfected undamaged or UV damaged pSP189 plasmids into Polη- or REV1-depleted 293T cells. The replicated plasmids were then used to perform traditional supF shuttle vector-based mutagenesis assay (TSMA) and “EasyMF” analysis. Both methods identified a significant difference in mutation frequency between undamaged and UV damaged plasmids extracted from either control, Polη or REV1 knocked down 293T cells (*p* < *0.05*) (Figs 2b and S2a,b). In the UV damaged samples, mutation frequencies measured by TSMA ranged from 7.55E-03 (siREV1-UV) to 1.55E-02 (siPolη-UV). Given that the mutation frequency measured by this approach was relative to the 95 bp supF marker gene, we could estimate the mutation frequency of single nucleotide in TSMA (TSMA-Sbase) based on a native model: e_L_ = 1-(1 − α)^L^, where e_L_ is the probability of having any change in a polynucleotide with length L, α is the average mutation rate per nucleotide. Using this model, the mutation frequencies of TSMA-Sbase in siNC-UV, siPolη-UV and siREV1-UV were estimated to be 1.11E-04, 1.64E-04, and 7.97E-05, respectively. Whilst, in the UV damaged samples, the mutation frequencies of entire plasmid measured by “EasyMF” ranged from 6.25E-05 (siREV1-UV) to 1.26E-04 (siPolη-UV). Meanwhile, the mutation frequencies of 95 bp target region measured by “EasyMF” ranged from 9.90E-05 (siREV1-UV) to 1.75E-04 (siPolη-UV). The statistical results indicated that the mutation frequency of entire plasmid or 95 bp target region measured by “EasyMF” was consistent with (no significant differences, *p*> 0.05) that measured by TSMA-Sbase.

To further verify the mutation frequency measured by “EasyMF”, we mixed phix174 DNA from two strains with separated genotypes (Table 1), at ratios of 100: 1, 1,000: 1, and 10,000: 1 for “EasyMF” libraries preparation. Sequencing results revealed that the “EasyMF” measured allele frequencies of two heterozygotes sites in all mixed samples were in accordance with the theoretical values (Table 2), suggesting that the “EasyMF” measured allele frequency could represent the true allele frequency.

Next, we analyzed minor allele frequency (MAF) based on the “EasyMF” results. We sampled 1 million mapped consensus reads of every different experimental replicates and calculated the MAF of every mutation. The statistic results demonstrated that over 99% MAF of all samples were lower than 0.001, in which 0 ~ 0.00001, 0.00001 ~ 0.0001 and 0.0001 ~ 0.001 dominated the results (Figs 2c and S3a,b). In addition, the proportion of MAFs with ranges of 0.00001 ~ 0.0001 and 0.0001 ~ 0.001 in UV damaged plasmids were higher than those of undamaged plasmids. These mutation frequencies were beyond the detection limitation of standard NGS approaches but not “EasyMF”.

We next compared the mutation frequencies of undamaged and UV damaged plasmids replicated from control, Polη or REV1 knocked down 293T cells to determine each mutation type changes induced by UV using “EasyMF”. From the sequencing data we can see the mutation frequencies of 10 types of mutation increased significantly *(p* < *0.05)* after UV damage in the examined cells, while the mutation frequencies of two other mutation types (C = >G, G = >C) had no obvious change *(p>* 0.05) (Fig. S4). Meanwhile, we also noticed that the mutation frequencies of C = >G and G = >C displayed large deviation among different experiments. We speculate that it might be caused by DNA damage of pSP189 resulting from high energy in the initial sonication step. To verify this, we used enzyme digestion instead of sonication to fragment plasmid DNA. As expected, the high mutation frequencies of these two mutation types were decreased and the large deviation was also diminished (Figs S5a–e and S6). In addition, the mutation frequencies of C = >G and G = >C also exhibited a significant increase (*p* < *0.05*), similar to other types of substitutions (Fig. S5 f) in UV-damaged samples. Therefore, the high mutation frequencies of C = >G and G = >C in plasmid pSP189 could mainly be attributed to the process of sonication (see *Discussion*). Therefore, in the following results, we excluded these two mutation types (C = >G and G = >C) to avoid the artificial error affecting the conclusions. Based on this, we calculated the mutation frequency of UV-damaged plasmid measured by “EasyMF” with enzyme digestion, the results showed siNC-UV has a mutation frequency of 9.18E-05, and siREV1-UV has a mutation frequency of 7.92E-05. These results were nearly identical to the mutation frequency measured by TSMA-Sbase and “EasyMF” with sonication, indicating that the mutation frequency measured by “EasyMF” could reflect the real mutation frequency.

As UV produces specific photoproducts at dipyrimidine sites in DNA, which will result in mutagenesis. We analyzed C = >T mutation frequencies at dipyrimidine and polypyrimidine sites specifically (Figs 2d,e and S7). For the UV-damaged plasmid DNA, the C = >T mutation frequencies at dipyrimidine and polypyrimidine sites were significantly increased compared to those at monopyrimidine sites in the control (p = 0.017 for dipyrimidine sites, p = 0.004 for polypyrimidine sites), Polη knocked down (p = 0.003 for dipyrimidine sites, p = 0.001 for polypyrimidine sites) or REV1 knocked down (p = 0.004 for dipyrimidine sites, p = 0.0002 for polypyrimidine sites) cells, respectively. For the undamaged plasmid DNA, the C = >T mutation frequencies at dipyrimidine and polypyrimidine sites were similar to those at monopyrimidine sites in the control (p = 0.630 for dipyrimidine sites, p = 0.427 for polypyrimidine sites), Polη knocked down (p = 0.627 for dipyrimidine sites, p = 0.417 for polypyrimidine sites) or REV1 knocked down (p = 0.795 for dipyrimidine sites, p = 0.568 for polypyrimidine sites) cells. These results indicate that “EasyMF” could dissect the mutation frequencies at different sequence contexts through its abundant sequencing data.

### Determination of TLS polymerases’ roles in UV-induced mutagenesis with “EasyMF”

**Figure 3:**
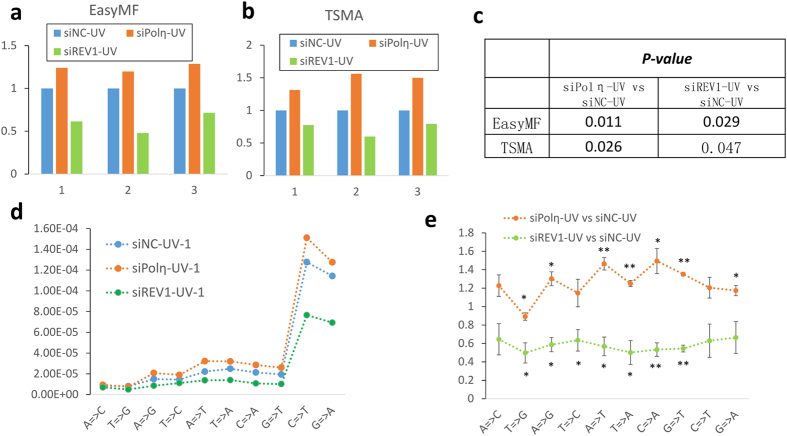
Mutation frequency changes in control, Polη or REV1 knocked down cells measured by “EasyMF” **(a)** and TSMA **(b)**. X axis represents different experimental replicates, while y axis represents the fold change of mutation frequency compared with corresponding siNC-UV. **(c)** The p-values of siPolη-UV versus siNC-UV and siREV1-UV versus siNC-UV. **(d)** The effects of Polη- or REV1-depletion on the base substitution frequencies of UV damaged pSP189 plasmid, one replicate was showed, another two were showed in Fig. S8. **(e)** The significance statistical analysis of base substitution of siPolη-UV and siREV1-UV compared with siNC-UV. Y axis is the fold changes of mutation frequency for each types of base substitutions.

Given its high sensitivity and low error rate, we then used “EasyMF” to dissect *in vivo* roles of Polη and REV1 in DNA damage-induced mutagenesis. The undamaged and UV damaged pSP189 plasmids were transfected into control, Polη and REV1 knocked down 293T cells followed by TSMA or “EasyMF”. Owing to the ultra-low mutation frequency changes, we took the mutation frequency fold change to distinguish *in vivo* roles of Polη and REV1 in UV-induced mutagenesis, with the mutation frequency of siNC-UV as baseline to diminish the variations among different experimental replicates. The “EasyMF” results revealed that the mutation frequency of UV damaged plasmids in control cells was significantly lower than that in Polη-depleted cells (p < 0.05), while it is remarkably higher than that in REV1-depleted cells (p < 0.05) (Fig. 3a,c). These trends of mutation frequency change were confirmed by TSMA (Fig. 3b,c). We further applied “EasyMF” method to determine mutational spectra of UV damaged supF shuttle vectors replicated in cells, which were either depletion of Polη or REV1 (Figs 3d and S8). Our data indicated the mutation frequencies of three mutation types (A = >T, T = >A, G = >T) exhibited an extremely significant (*p* < *0.01*) increase, with three types (A = >C, C = >T, T = >C) were insignificantly (*p*>*0.05*) increased, and three mutation types (A = >G, C = >A, G = >A) were significantly (*0.01* < *p* < *0.05*) increased in the Polη knocked down 293T cells, surprisingly, there was one mutation type (T = >G) decreased. Whilst, the mutation frequencies of two mutation types (G = >T, C = >A) showed an extremely significant (*p* < *0.01*) decrease, five mutation types (T = >G, A = >G, T = >C, T = >A, A = >T) were significant (*0.01* < *p* < *0.05*) decreased, and three types (A = >C, C = >T, G = >A) were insignificantly *(p>0.05)* decreased in the REV1 knocked down 293T cells (Fig. 3e).

### High reproducibility and sensitivity of the “EasyMF” for mutagenesis analysis

**Figure 4:**
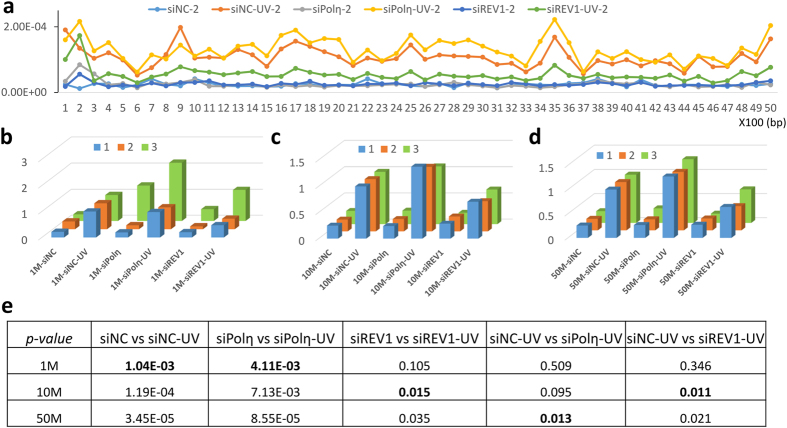
Reproducibility and sensitivity of the “EasyMF”. **(a)** Overview of mutation frequency in pSP189 plasmid, slide window size is 100bp. X axis is the genome position, y axis is the mutation frequency of each region. **(b–d)** Fold changes of mutation frequency relative to corresponding siNC-UV from sub-sampled 1 Mb, 10 Mb and 50 Mb data, 3 replicates were plotted. **(e)** *p-values* of the mutation frequency difference between undamaged and UV damaged plasmids in indicated circumstances, or siPolη-UV and siNC-UV, or siREV1-UV and siNC-UV in 1 Mb, 10 Mb and 50 Mb sub-sampled data.

To further explore the mutation frequency distribution across the whole pSP189 plasmid, we split the genome into 50 small regions with 100 bp slide window and calculated the mutation frequency separately. We found that most regions shared a similar trend in UV-induced mutation frequency changes—increased in Polη-depleted cells and decreased in REV1-depleted cells (Figs 4a and S9). In addition, results from different experimental replicates indicated these mutation frequency patterns were stable and highly repeatable (Fig. S9). However, the “EasyMF” results showed that the mutation frequency varied in different small regions and there were mutation hotspots (such as region 9, 17, 25, 35) and conserved regions (6, 15, 37) in the pSP189 plasmid, which suggests that certain selection pressure may affect the mutation frequency of different regions. We picked up one conserved region (region 37) to explore why its mutation frequency is lower than the average level. Out of curiosity, we found the essential elements for pSP189 replication in 293T cells–promoter and origin of SV40—did exist in this region. It indicates that selection pressure bring down the observed mutation rate, for lacking of progeny. However, the function of hotspot regions (such as 17, 35) looks not so indispensable^34^.

To reduce the data volume and sequencing cost, we assessed how much data is enough for sufficiently inferring the results. Here, we introduce two terms—circularization rate (CR) and cycle mapping rate (CMR)—to characterize the sequencing data. CR means the percentage of consensus reads were detected in raw PE reads, whilst, CMR means the percentage of consensus reads that can be mapped into the plasmid reference. Based on the “EasyMF” sequencing data, our sonication samples had the average CR of 73% (±5.7%), and the average CMR of 35% (±8.3%) (Table S1). Under such circumstances, we then randomly sampled 1 Mb, 10 Mb, 50 Mb, 100 Mb and 500 Mb data three times from each sample, and the average mutation frequency of three sampled data was used for assessment. The sub-sampled results showed even 1 Mb data was adequate to distinguish the mutation frequency difference between undamaged and UV damaged plasmid in control and Polη knocked down cells, while 10 Mb data would be needed in REV1 knocked down cells (Figs 4b–d and S10). To dissect the *in vivo* roles of TLS polymerases in DNA damage-induced mutagenesis, we compared the measured mutation frequency of siNC-UV to siPolη-UV, and siNC-UV to siREV1-UV in 1 Mb, 10 Mb, 50 Mb, 100 Mb and 500 Mb sub-sampled data separately. The results indicated 50 Mb data would be required to distinguish siPolη-UV from siNC-UV, and 10 Mb data was sufficient to distinguish siREV1-UV from siNC-UV (Figs 4b–d and S10). Therefore, less than 100 Mb “EasyMF” data was quite enough to study the effects of TLS polymerases in mutagenesis, which means the sequencing cost would be ultra-low for each sample.

## Discussion

The supF mutation assay is widely employed to examine the effects of lesion bypass DNA polymerases on damaged-induced mutagenesis. Since only surviving clones are counted for mutation rate calculation, the mutagenesis detection is quite restricted. Besides, it is quite laborious and time consuming, with limited information be obtained. To avoid these blemishes, we developed an updated USNGS approach “EasyMF”, through which we figured out the mutation frequency of UV (220 J/m^2^) damaged pSP189 plasmid in control cells was around 1.0E-04, with 99% MAF were lower than 0.1%. We found that mutation frequencies of all possible mutation types (n = 12) increased significantly *(p* < *0.05)* after UV damage. Remarkably, the “EasyMF” results clearly revealed that the mutation frequency of UV damaged plasmid was significantly increased in Polη-depleted cells (*p* < *0.05*), and dramatically reduced in REV1-depleted cells (*p* < *0.05*) compared with that in control cells. The “EasyMF” results also indicated there were some mutation hotspots and conserved regions existed in the pSP189 plasmid. Our sampling sequencing data demonstrated 10 Mb data was sufficient for distinguishing the difference in mutation frequency between undamaged and UV damaged plasmids, whilst, 50Mb data would be needed for detecting the influences of REV1 and Polη on UV-induced mutagenesis.

“EasyMF” establishes a whole package of experimental and data processing pipelines to efficiently dissect the *in vivo* roles of TLS polymerases in DNA damage-induced mutagenesis. Following our described pipelines, the experiments (“EasyMF” libraries preparation) can be finished in about three days initiating from the purified samples. The sequencing period mainly depends on the sequencing platform, for example, less than one day for Illumina Miseq, 3 ~ 5 days for HiSeq 2500, and 10 ~ 14 days for HiSeq 2000. According to our sampling sequencing data, 100Mb data is sufficient (tested on HiSeq 2000, HiSeq2500), that implied the sequencing cost would be ultra-low. For data analysis, only less than 1 h would be needed to get the mutation frequency of each mutation type from the original fastq data (~1G) using our pipelines. This improved approach will allow us to conveniently obtain more comprehensive and representative information.

In our study, we chose Polη and REV1 as target proteins to prove the effective application of “EasyMF”. UVC-induced mutations are mainly targeted to dipyrimidine sites containing cytosines and C to T transitions dominated the mutation spectrum at dipyrimidine sites. The suppression of Polη could cause a significant increase in the frequency of mutations, with a predominant C to T mutation^10^. REV1 has been reported to be required for induced mutagenesis by physical and chemical agents in mammalian cells, with siRNA-mediated reduction of REV1 decreasing the UV-induced mutation frequencies^35^,^36^,^37^. The mutation frequency of pSP189 plasmid (220 J/m^2^ UVC damaged) in Polη knocked down cells is significantly increased than that in the control cells (*p* = 0.022). While the mutation frequency of pSP189 plasmid in REV1 knocked down cells is significantly lower than that in the control cells (*p* = 0.027), in line with the previous studies^10^,^35^,^36^,^37^. Moreover, based on abundant sequencing data, we have illustrated the mutation frequencies of 12 different mutation types and C = >T mutation frequencies at the dipyrimidine sites caused by Polη and REV1 depletion, which will be helpful to understand their functions in UV mutagenesis, tumor initiation and progression.

Although “EasyMF” could detect low frequency mutations, it still has some limitations, especially the artificial DNA damage before RCA could not be distinguished from the genuine mutation. As the shearing condition could increase error rate directly, it is important to keep DNA from damage to the most extent. Especially, the temperature should not exceed 4 °C, and the plasmid DNA should be resuspended using Buffer AE (10 mM Tris-Cl, 0.5 mM EDTA, pH 9.0) or TE (10 mM Tris-HCl, 1 mM EDTA, PH 8.0) instead of H_2_O. Even though, the sonication fragmentation method still resulted in certain degree of background error rates in pSP189 plasmid for two types of mutation (C = >G and G = >C). To validate this conclusion, we performed tens of experiments to prepare “EasyMF” libraries of the plasmid DNA extracted from 293T cells by sonication and enzyme digestion, respectively. The results showed mutation frequencies of C = >G, G = >C, G = >A and C = >T were higher than other 8 mutation types (Fig. S6 a) in 21 sonication sequencing data. In contrast, only G = >A and C = >T mutation frequencies were higher than other mutation types (Fig. S6 b) in 10 enzyme digestion sequencing data. According to this result, we speculated that sonication results in the higher G = >C and C = >G mutations. However, we didn’t find the higher G = >C and C = >G mutations (Fig. S6c) when we used the sonication method to detect the mutation frequency of *E.coli* genome, this result was in line with a previous study^22^, in which a similar method was tested in yeast genome. Therefore, it is likely the higher G = >C and C = >G mutations were the consequences of a combination of sonication and pSP189 plasmids, which awaits for further studies. All these results suggest the enzyme digestion method will be a better choice when the samples are sufficient, but sonication method is still a prior choice based on its stability and high recovery rate when samples are below 200 ng.

In summary, we have established an improved experimental and data processing platform for the rapid analysis of mutations on the supF shuttle vector in mammalian cells. We believe this powerful approach will allow us to conveniently screen regulators of TLS pathway and ultimately provide insight into the mechanisms of genome stability and mutagenesis. Furthermore, our experimental protocol and analysis pipeline could be easily used in other applications of detecting ultra-low frequency mutations.

## Materials and Methods

### Cell culture and RNA interference

Human 293T cells was obtained from the American Type Culture Collection (Rockville, MD). The cells were grown in DMEM medium supplemented with 10% fetal bovine serum at 37 °C in the presence of 5% CO_2_ if not specified.

siRNAs directed against REV1 and Polη were obtained from GenePharma (Shanghai, China). The gene-specific target sequences were as follows: REV1 (GAACAGUGACGCAGGAAUA)^38^, and Polη (CAGCCAAATGCCCATTCGCAA)^39^. The negative control siNC sequence (UUCUCCGAACGUGUCACGU) was obtained from GenePharma. The introduction of small interfering RNA (siRNA) into cells was carried out with RNAiMAX (Invitrogen). Briefly, cells were exposed to 100 nM siRNA overnight in the presence of serum, followed by a change in medium the next morning. Whole cell lysates were harvested at 72 h after siRNA transfection. Western blot was used to validate the knockdown efficiencies of these siRNAs.

### supF shuttle vector-based mutagenesis assay

Mutation frequencies were measured using the supF shuttle vector system as described previously^9^, which measures TLS activity in mammalian cells^10^,^40^. 293T cells were transfected with the indicated siRNAs twice. 48 h after the first transfection, cells were transfected with an undamaged or UVC-irradiated (220 J/m^2^) pSP189 reporter plasmid. 48 h later, the pSP189 plasmid was retrieved from the 293T cells using a DNA miniprep kit (Tiangen). The purified plasmid was digested with DpnI and separated into two parts. One was purified to perform “EasyMF” (see “**EasyMF**” **library preparation**), whilst the other one was transformed into the MBM7070 bacterial strain. The transformed MBM7070 cells were grown on Luria–Bertani plates containing 200 μM isopropy β-D-1-thiogalactopyranoside, 100 μg/ml X-gal, and 100 μg/ml ampicillin. The mutation frequency in the supF coding region was determined by enumerating the ratios of blue (wile-type) and white (mutant) colonies. The pSP189 plasmid and MBM7070 strain were gifts from Dr. M. Seidman^41^.

### Western blotting

72 h after the first siRNA transfection, 293T cells were harvested and lysed directly in RIPA buffer (50 mM Tris-HCl [pH 7.4], 150 mM NaCl, 1 mM EDTA, 1% Triton X-100, 1% Sodium deoxycholate (DOC), 0.1% SDS) supplemented with protease inhibitor mix (Sigma). The supernatants were separated by SDS-PAGE and detected by immunoblotting with antibodies against either REV1 (Rabbit polyclonal antiserum against REV1_872–1150_ was made by Covance)^42^, Polη (Abcam17725), or β-tubulin (Beijing Protein Innovation).

### “EasyMF” library preparation

Purified plasmid DNA ( ~ 200 ng) was sheared into 100 bp fragments in Buffer AE (10 mM Tris-Cl, 0.5 mM EDTA) using Covaris S220 in 130 μl volume (shearing condition: duty cycle: 10%, intensity: 5, cycles per burst: 100, time: 600 s, temperature <4 °C), then purified with Oligo Clean & concentrator kit (Zymo, D4060). The purified DNA was phosphorylated at 37 °C for 30 min in a reaction consisting of 22 μl DNA, 0.5 μl T4 PNK (T4 Polynucleotide Kinase, NEB, M0201S), 2.5 μl T4 DNA ligase buffer with 10 mM dATP, then was run on a 4% agarose gel at 80 V for 70 min. Subsequently, the gel regions with DNAs in length of 60 ~ 100 bp marked by the 20 bp DNA ladder (Takara) were specifically cut out, and further extracted using QIAGEN MinElute Gel Extraction Kit (6X buffer QG). Alternatively, if the plasmid DNA less than 100 ng, the fragmentation step should be performed in the following shearing conditions: duty cycle: 10%, intensity: 5, cycles per burst: 100, time: 900 s, temperature <4 °C, and purified with MinElute Reaction Cleanup Kit (3X buffer ERC) to remove small fragments (less than 70 bp). Purified DNA was phosphorylated using T4 PNK as above described, then purified with Oligo Clean & concentrator kit (Zymo, D4060). The ultimate DNA concentration was calibrated using Qubit 2.0 dsDNA HS Assay kit.

The size-selected and purified DNA was concentrated to 12 μl, then denatured at 95 °C for 3 min followed by incubation on ice for 3 min. Then the sample was supplemented with a mixture of 10X Cirligase buffer (1.5 μl), 50 mM MnCl_2_ (0.75 μl), Cirligase (0.75 μl) (Epicentre CL9025K) and further incubated at 60°Cfor 14 h before the reaction was stopped by heating at 80 °C for 10 min. Subsequently, 0.75 μl Exonuclease I (NEB, M0293S) and 0.75 μl Exonuclease III (NEB, M0206S) were added into the reaction and jointly incubated at 37 °C for 1 h. The enzymes were inactivated at 80 °C for another 20 min. The successfully circularized DNA was purified using the Oligo Clean & concentrator kit (Zymo, D4060).

The circularized DNA was concentrated to 1 μl, then mixed with 9 μl Sample buffer (illustra GenomiPhi V2 DNA Amplification Kit, GE Healthcare, 25-6600-30). The total 10 μl mixture was denatured at 95 °C for 3 min and left on ice immediately for another 3 min before incubation with 9 μl Reaction buffer (illustra GenomiPhi V2 DNA Amplification Kit, GE Healthcare, 25-6600-30), 1 μl of Enzyme Mix (illustra GenomiPhi V2 DNA Amplification Kit, GE Healthcare, 25-6600-30), 1 μl UDG (uracil-DNA glycosylase, NEB, M0280S), 1μl Fpg (formamidopyrimidine DNA glycosylase, NEB, M0240S) at 30 °C for 35 ~ 65 min. The reaction was stopped by incubation at 65 °C for 10 min when the amplification product reached to 0.5 ~ 1 μg (monitored by Qubit). The product was then purified with 1X Ampure XP beads. The recovered DNA can be used for preparing standard NGS libraries.

### Standard NGS library preparation

The amplified DNA ( ~ 800 ng) was sheared into 700 bp in ddH_2_O (85 μl) using Covaris S220 (shearing condition: duty cycle: 5%, intensity: 3, cycles per burst: 200, time: 75 s), then end-repaired using NEBNext® End Repair Module (NEB, E6050S) and purified with 1X Ampure XP beads. The NEBNext® dA-Tailing Module (NEB, E6053S) was used for dA-tailing. 1X Ampure XP beads were used to clear up the reaction mixture. Ligation was performed at 20 °C for 30 min using NEBNext® Quick Ligation Module (NEB, E6056S) and 1 μl barcode adaptor (Bioo Scientific, NEXTflex™ DNA Barcodes, 514102). The production was purified with 1X Ampure XP beads and run on a 2% agarose gel to perform the size-selection. The gel with DNA in length of 400 ~ 700 bp was cut out and further extracted using QIAGEN MinElute Gel Extraction Kit (3X buffer QG). The ultimate DNA concentration was calibrated using Qubit 2.0 dsDNA HS Assay kit. PCR was performed in a reaction consisting of 24 μl ligated DNA (~30 ng) in ddH_2_O, 1 μl NEXTflex™ Primer Mix (Bioo Scientific, 514102), 25 μl KAPA HiFi HotStart ReadyMix (2X) as the following cycling conditions: 98 °C for 45 s and 10 cycles of 98 °C for 15 s, 65 °C for 30 s, 72 °C for 1 min, then 72 °C for 4 min and held at 4 °C. The production was purified with 1X Ampure XP beads and run on a 2% agarose gel, the gel with DNA in length of 400 ~ 700bp was cut out and extracted using QIAGEN MinElute Gel Extraction Kit. The purified DNA then be used to high through put DNA sequencing (such as Illumina HiSeq 2000, HiSeq 2500).

### “EasyMF” Data Processing

The data processing of “EasyMF” reads is different from that of regular re-sequencing data. First, a consensus sequence should be determined from multiple tandem copies of circular DNA within a pair of PE reads. Second, the break point of circularized DNA should be accurately detected by mapping it to the reference genome.

The consensus sequences (CSs) can be determined by aligning Read1 and Read2 from the same pair of PE reads with a mismatch rate of approximately 0.05. The detailed procedure of CSs determination was summarized as following. Firstly, the Read1 and the reverse complement of Read2 are literally merged into one longer read according to their maximum of overlapped bases. Secondly, the merged read is aligned against itself to find the smallest unit of tandems, called CS. The base content of CS comes from one copy of tandems. The base qualities of CS is calculated by summing original base qualities in sequencing, but with the maximum limit at phred score of 93. The base quality will be set to minimum at a) sites be passed by less than twice, and b) sites be passed with different base content.

The CSs were mapped onto the reference genome using Bwa^43^. Since every position on the CSs could be the correct junction of the linear DNA sequence, to search the most possible junction, we should try to start to read the sequences from every position. In practise, our program has a tradeoff between mapping time and CS length by binning positions of CS. Defaultly, we reduce the mapping time into 1/5 by discarding one bin of five bases.

The source code of “EasyMF” data processing is available on:

https://sourceforge.net/projects/easymf/files/

In this study, all statistical tests were performed with two tailed student t test, of which the alpha level is 0.05.

## Additional Information

**Accession code**: All raw sequencing data collected for this study have been deposited in the NCBI Sequence Read Archive (SRA) repository, [http://www.ncbi.nlm.nih.gov/sra] under accession number: PRJNA302825.

**How to cite this article**: Wang, K. *et al.* Using ultra-sensitive next generation sequencing to dissect DNA damage-induced mutagenesis. *Sci. Rep.* **6**, 25310; doi: 10.1038/srep25310 (2016).

## Supplementary Material

Supplementary Information

## Figures and Tables

**Table 1:** Polymorphic sites of two phage phix174.

Chr	Pos	Ref	Alt	Promega_AF		NEB_AF	
phix174	3111	G	A	0.311187	0.688813	1	0
phix174	3133	C	T	0.798669	0.201331	0	1

Chr: Chromosome, Pos: Chromosome position, Ref: Reference allele, Alt: Alternate allele, AF: allele frequency. Phix174 DNA were purchased from the commercial companies [Promega(cat: D1531) and NEB(cat: N3021S)], then sequenced them about 200,000X using standard NGS method on Illumina hiseq2500 platform separately to call fixed variants. The allele frequency of each strain was calculated according to the support reads number of each sites, and only two polymorphic sites (3111, 3133) were found which were different between these two strains.

**Table 2:** The theoretical and measured allele frequency of mixed phix174 samples at ratios of 100: 1, 1,000: 1, and 10,000: 1.

NEB ~ Promega	100 ~ 1		1000 ~ 1		10000 ~ 1	
Phix174: 3111	G	A	G	A	G	A
Theoretical	0.993168	6.83E-03	0.999311	6.89E-04	0.999931	6.9E-05
Measured	0.994877	5.12E-03	0.999759	2.41E-04	0.999956	4.44E-05
Phix174: 3133	C	T	C	T	C	T
Theoretical	7.92E-03	0.992079	7.99E-04	0.999201	8E-05	0.999920
Measured	6.70E-03	0.993297	4.03E-04	0.999597	3.67E-05	0.999963
