# Supplementary material for: Using ultra-sensitive next generation sequencing to dissect DNA damage-induced mutagenesis
Source: Sci Rep. 2016 Apr 28;6:25310. doi: 10.1038/srep25310 (PMC4848531; doi:10.1038/srep25310)
Supplement: Supplementary Information [file srep25310-s1.pdf]

Supplementary Information

Using ultra-sensitive next generation sequencing to dissect DNA damage-induced mutagenesis

Kaile Wang[1,2*], Xiaolu Ma[1,2*], Xue zhang[1,2], Dafei Wu[1], Chenyi Sun[1,2], Yazhou Sun[1,2], Xuemei Lu[1], Chung-I Wu[1,3,4*], Caixia Guo[1*], Jue Ruan[1,5*]

Sonication fragmentation						
Samples	Data(G)	# of reads	# of CRs	# of mapped reads	CR	CMR
siNC-1	0.86	4,238,808	3,151,650	1,131,063	74%	36%
siNC-2	0.98	4,855,449	3,279,203	1,106,520	68%	34%
siNC-3	1.03	5,083,247	3,338,320	1,085,851	66%	33%
siNC-UV-1	1.10	5,467,023	3,938,378	1,499,781	72%	38%
siNC-UV-2	1.06	5,268,303	3,945,980	1,279,668	75%	32%
siNC-UV-3	2.25	9,006,083	7,474,278	2,544,592	83%	34%
siPolη-1	1.26	6,251,923	4,482,327	1,962,222	72%	44%
siPolη-2	1.00	4,928,787	3,592,257	1,435,503	73%	40%
siPolη-3	1.04	5,170,514	3,508,162	1,007,181	68%	29%
siPolη-UV-1	0.83	4,111,644	3,010,474	1,111,081	73%	37%
siPolη-UV-2	1.28	6,331,659	4,870,025	1,683,750	77%	35%
siPolη-UV-3	1.15	5,679,177	3,935,243	1,026,395	69%	26%
siREV1-1	1.12	5,540,089	3,779,552	1,402,063	68%	37%
siREV1-2	0.88	4,336,277	3,177,523	1,772,043	73%	56%
siREV1-3	2.33	9,313,185	7,724,635	1,288,637	83%	36%

siREV1-UV-1	1.07	5,311,304	4,279,102	1,705,834	81%	40%
siREV1-UV-2	1.33	6,572,818	4,911,613	2,025,567	75%	41%
siREV1-UV-3	2.06	8,251,040	6,751,050	1,819,970	82%	45%
Enzyme digestion method (random fragmentation)						
siNC-E	0.98	4,850,938	3,722,827	1,084,254	77%	29%
siNC-UV-E	1.09	5,409,989	4,574,330	1,187,518	85%	26%
siREV1-E	0.96	4,738,271	3,934,043	2,257,145	83%	57%
siREV1-UV-E	1.24	6,130,421	5,112,568	2,265,001	83%	44%
Double restriction endonucleases digest for 95bp target region						
siNC_95-E	3.20	15,821,938	8,576,452	7,155,368	54%	83%
siNC-UV_95-E	5.19	25,677,530	16,713,140	14,539,557	65%	87%
siPolη_95-E	3.53	17,465,615	8,728,740	5,026,414	50%	58%
siPolη-UV_95-E	2.68	13,260,260	3,254,456	1,397,343	25%	43%
siREV1_95-E	2.93	14,529,637	8,616,862	6,524,684	59%	76%
siREV1-UV_95-E	3.67	18,149,949	10,145,964	7,490,043	56%	74%

Supplementary Table S1. Samples and basic sequencing data. Different DNA fragmentation approaches (sonication, random enzyme digestion or restriction endonucleases digestion) were employed to fragment the original plasmid DNA. CRs: consensus reads, CR: circularization rate, CMR: cycle mapping rate.

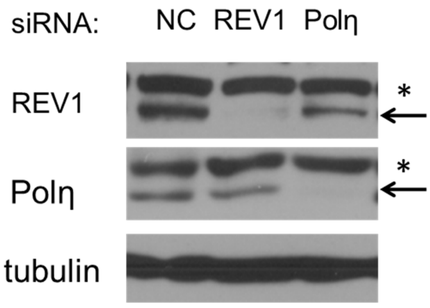

Supplementary Fig. S1 Western blotting showing the knockdown efficiencies of siREV1 and siPolη in 293T cells. 293T cells were transfected with siNC or siREV1 or siPolη. 72 h later, the

whole cell extracts were harvested. The expressions of REV1 and Polη were examined through western blotting using antibodies against REV1 or Polη, respectively. Tubulin: loading control. * represents non-specific bands.

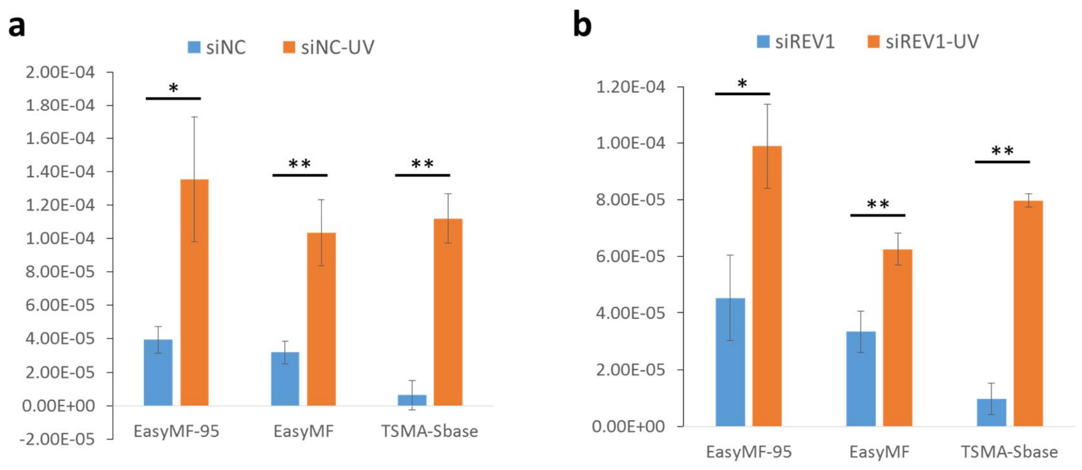

Supplementary Fig. S2 Mutation frequencies in undamaged and UV damaged pSP189 plasmids were determined after they were recovered from siRNA-treated 293T cells through indicated approaches. siNC and siNC-UV represent undamaged or UV damaged plasmid transfected into control 293T cells, respectively; siREV1 and siREV1-UV represent undamaged or UV damaged plasmid transfected into REV1 knocked down 293T cells, respectively. *: 0.01=< p< 0.05, **: p< 0.01.

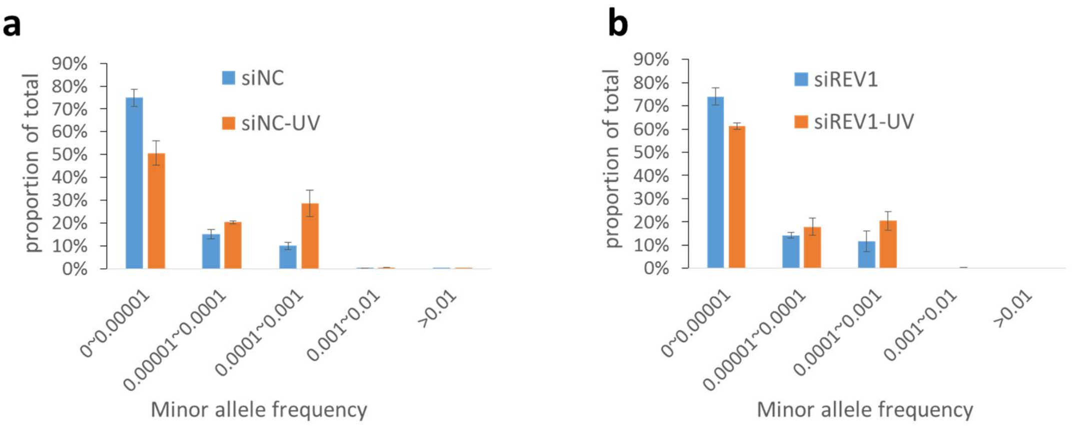

Supplementary Fig. S3 Distribution of MAFs of undamaged and UV damaged pSP189 plasmid in different host cells (control and siREV1-treated 293T cells).

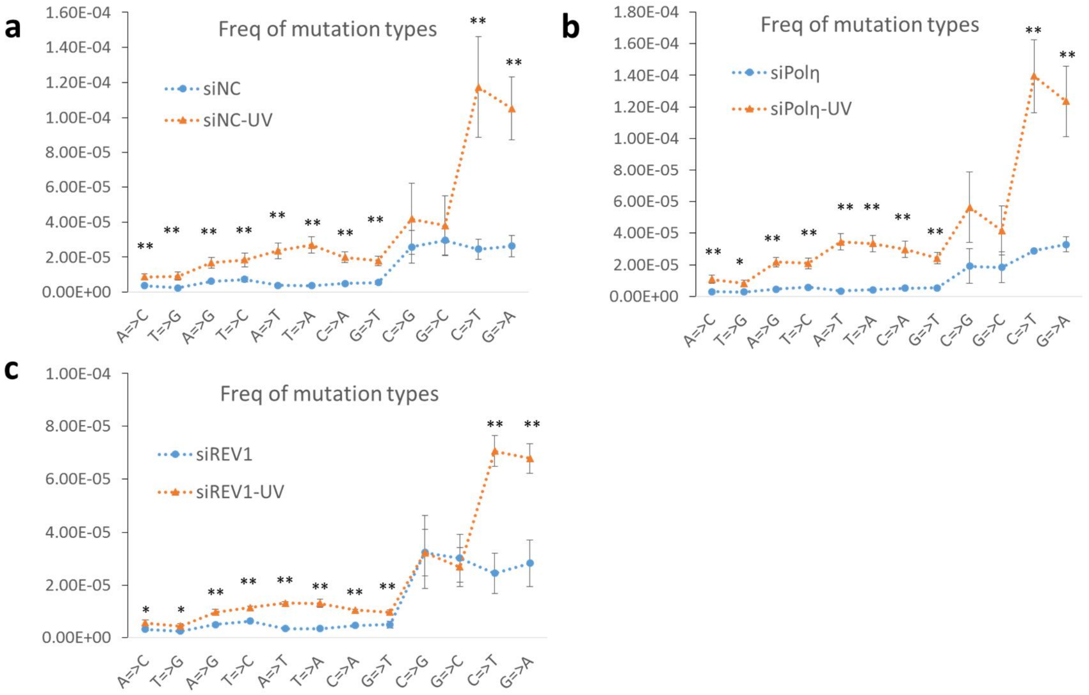

Supplementary Fig. S4 The mutation frequency of different mutation types on the indicated plasmids in control **(a)**, Polη **(b)** or REV1 **(c)** knocked down cells. Sonication methods were taken to fragment the plasmid DNA for single strand ligation.

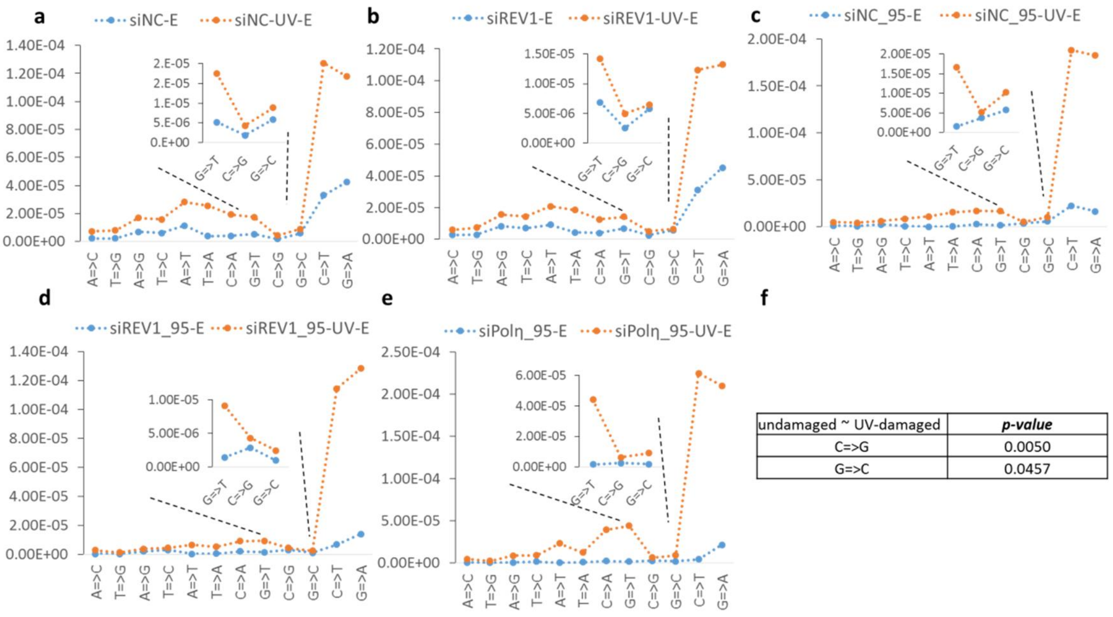

undamaged ~ UV-damaged	*p-value*
C=>G	0.0050
G=>C	0.0457

Supplementary Fig. S5 The mutation frequencies of different mutation types. **(a, b)** Random fragmentation method through an enzymatic digestion were used to fragment the plasmid DNA prior to single strand ligation. **(c-e)** The 95-bp target region was cut out by using two restriction endonucleases and enriched for single strand ligation. **(f)** Depiction of the mutation

frequencies two types of mutation (C=>G, G=>C) in UV damaged and undamaged plasmids measured by enzyme digested based “EasyMF”.

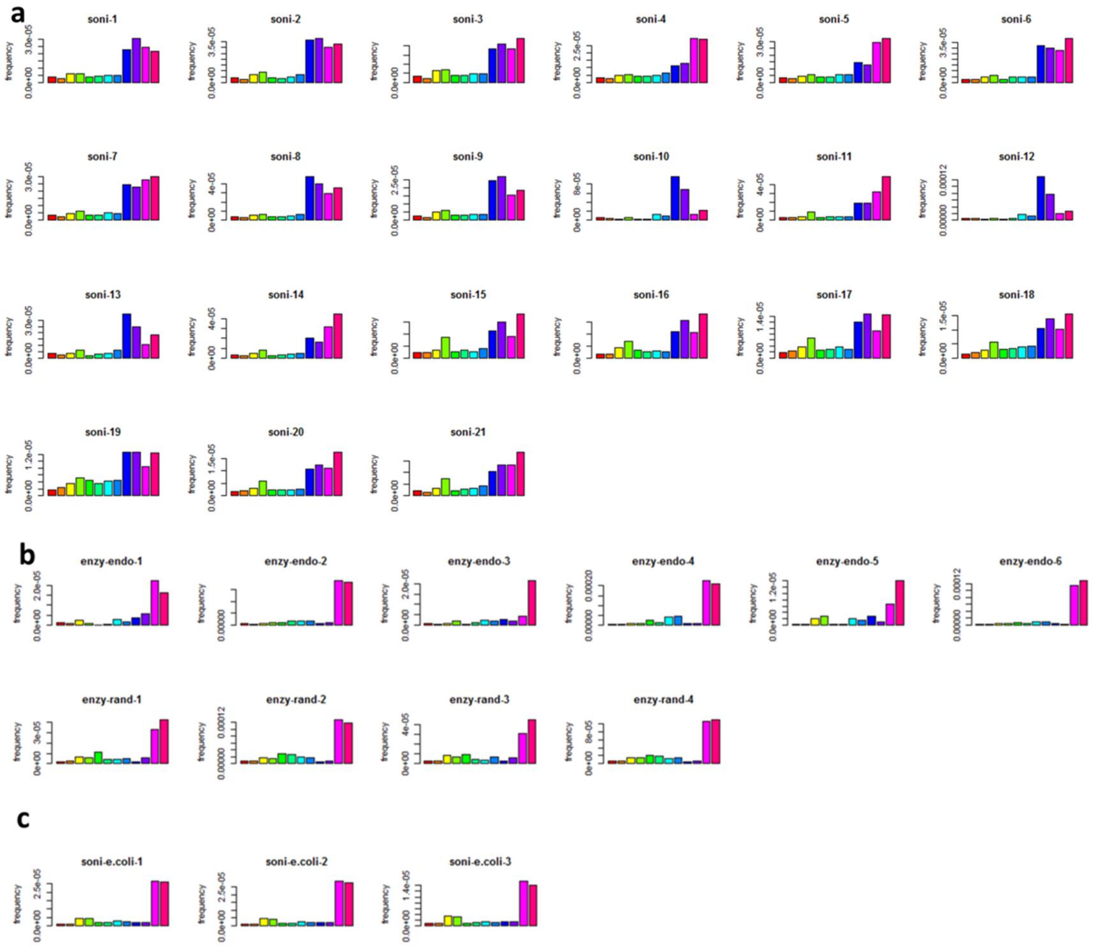

Supplementary Fig. S6 12 kinds of mutation type’s mutation frequencies of undamaged plasmid in host cells. 12 bars in each sub-figure represent 12 mutation types, from left to right are: A=>C, T=>G, A=>G, T=>C, A=>T, T=>A, C=>A, G=>T, C=>G, G=>C, C=>T, G=>A. The C=>G and G=>C are displayed with dark-blue and purple separately. **(a)** 21 independent experiments, in which sonication was used to fragment pSP189 DNA, were plotted. **(b)** 10 independent experiments, in which endonucleases (the first six sub-figures) or random digestion enzymes (the last four sub-figures) were used to fragment pSP189 DNA, were plotted. **(c)** 3 independent experiments, in which sonication was used to fragment *E.coli* DNA, were plotted.

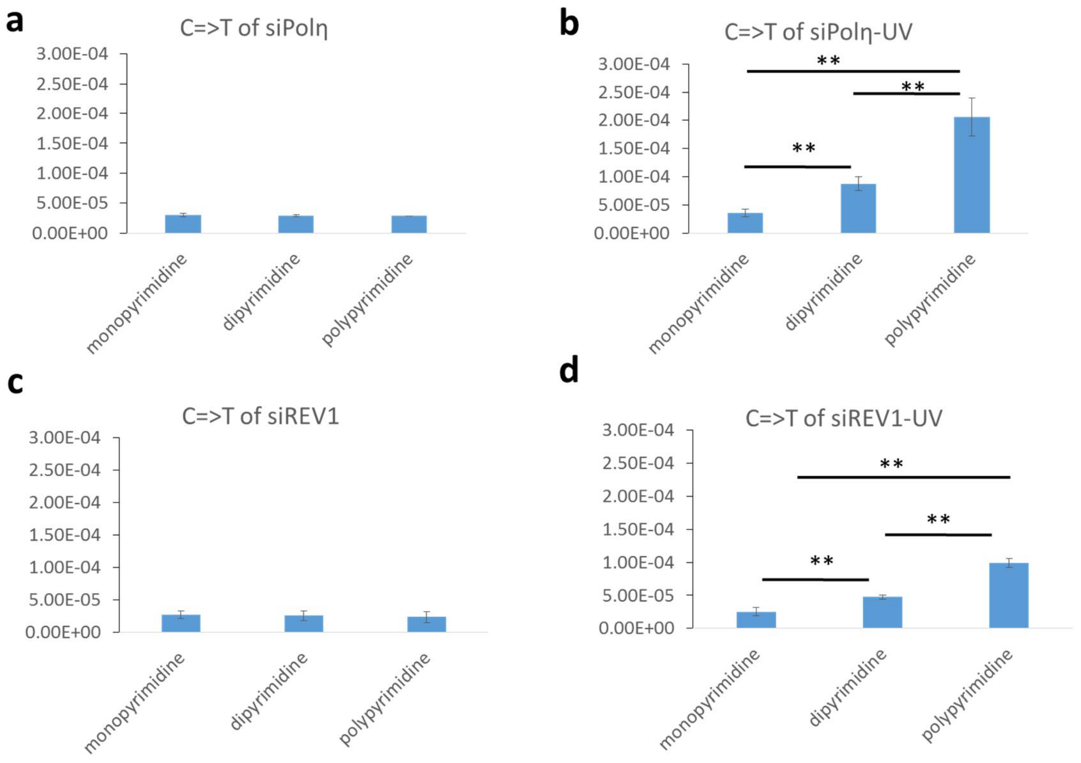

Supplementary Fig. S7 The C=>T mutation frequencies at monodipyrimidine, dipyrimidine and polypyrimidine sites from Polη and REV1 knocked down 293T cells. **(a, c)** C=>T mutation frequencies at monodipyrimidine, dipyrimidine and polypyrimidine sites of undamaged plasmid in Polη and REV1 knocked down 293T cells. **(b, d)** C=>T mutation frequencies at monodipyrimidine, dipyrimidine and polypyrimidine sites of UV damaged plasmid in Polη and REV1 knocked down 293T cells.

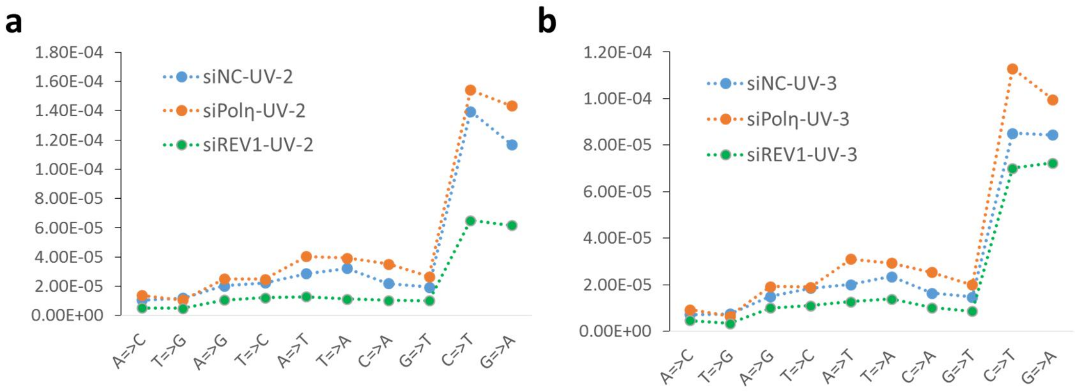

Supplementary Fig. S8 The effects of Polη- or REV1-depletion on the base substitution frequencies of UV damaged pSP189 plasmid. **(a, b)** Results from other two independent experiments were showed.

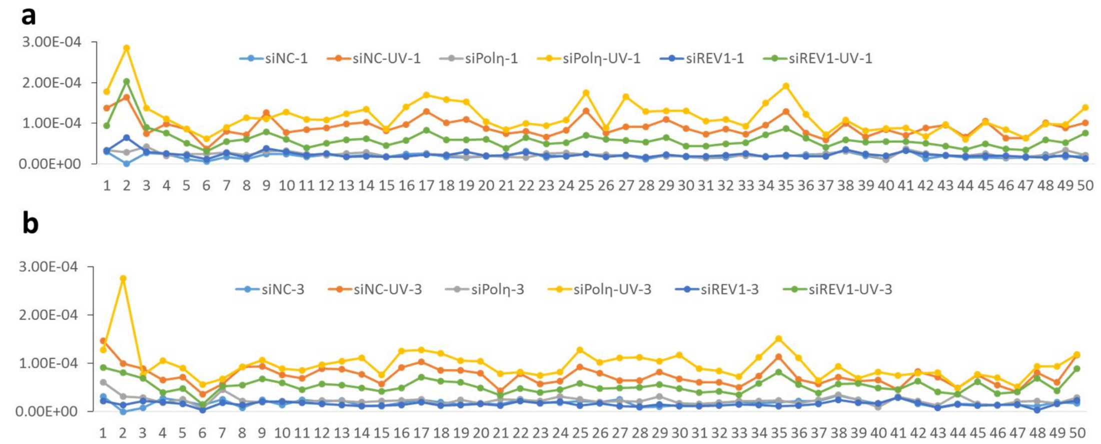

Supplementary Fig. S9 Pattern of mutation in the whole pSP189 plasmid by “EasyMF” approach. Slide window size is 100 bp. X axis is the genome position. y axis is the mutation rate. Results from two individual experiments were showed.

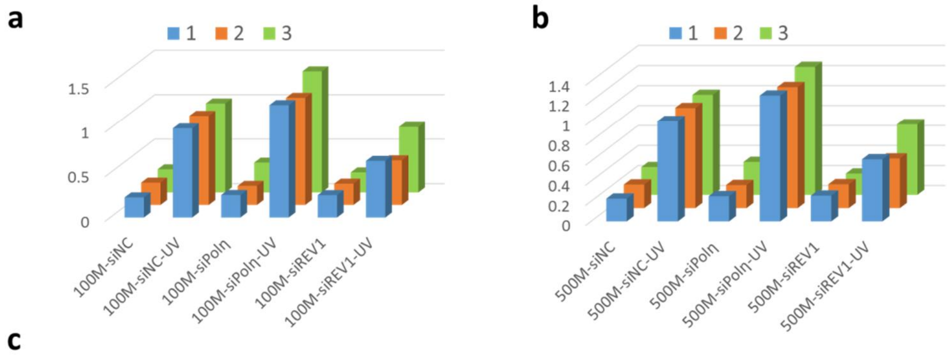

c

p-value	siNC vs siNC-UV	siPolη vs siPolη-UV	siREV1 vs siREV1-UV	siNC-UV vs siPolη-UV	siNC-UV vs siREV1-UV
100M	1.74E-04	1.11E-04	0.033	0.027	0.031
500M	4.22E-04	2.65E-04	0.032	6.78E-03	0.023

Supplementary Fig. S10 (a, b) Fold changes of mutation frequencies in indicated circumstances relative to corresponding siNC-UV from random sampling of 100M and 500M data. **(c)** p-values of the difference between UV damaged and undamaged, between siPolη-UV and siNC-UV, and between siREV1-UV and siNC-UV.
